# Moderators of wellbeing interventions: Why do some people respond more positively than others?

**DOI:** 10.1371/journal.pone.0187601

**Published:** 2017-11-06

**Authors:** R. Adele. H. Wang, S. Katherine Nelson-Coffey, Kristin Layous, Katherine Jacobs Bao, Oliver S. P. Davis, Claire M. A. Haworth

**Affiliations:** 1 School of Experimental Psychology, University of Bristol, Bristol, United Kingdom; 2 MRC Integrative Epidemiology Unit, University of Bristol, Bristol, United Kingdom; 3 School of Social and Community Medicine, University of Bristol, Bristol, United Kingdom; 4 Department of Psychology, Sewanee: The University of the South, Sewanee, Tennessee, United States of America; 5 Department of Psychology, California State University, East Bay, Hayward, California, United States of America; 6 Department of Psychology, Manhattanville College, Purchase, New York, United States of America; Jilin University, CHINA

## Abstract

Interventions rarely have a universal effect on all individuals. Reasons ranging from participant characteristics, context and fidelity of intervention completion could cause some people to respond more positively than others. Understanding these individual differences in intervention response may provide clues to the mechanisms behind the intervention, as well as inform future designs to make interventions maximally beneficial for all. Here we focus on an intervention designed to improve adolescent wellbeing, and explore potential moderators using a representative and well-powered sample. 16-year old participants (*N* = 932) in the Twins Wellbeing Intervention Study logged online once a week to complete control and wellbeing-enhancing activities consecutively. Throughout the study participants also provided information about a range of potential moderators of intervention response including demographics, seasonality, personality, baseline characteristics, activity fit, and effort. As expected, some individuals gained more from the intervention than others; we used multi-level modelling to test for moderation effects that could explain these individual differences. Of the 15 moderators tested, none significantly explained individual differences in intervention response in the intervention and follow-up phases. Self-reported effort and baseline positive affect had a notable effect in moderating response in the control phase, during which there was no overall improvement in wellbeing and mental health. Our results did not replicate the moderation effects that have been suggested by previous literature and future work needs to reconcile these differences. They also show that factors that have previously been shown to influence baseline wellbeing do not also influence an individual’s ability to benefit from a wellbeing intervention. Although future research should continue to explore potential moderators of intervention efficacy, our results suggest that the beneficial effect of positive activities in adolescents were universal across such factors as sex and socioeconomic status, bolstering claims of the scalability of positive activities to increase adolescent wellbeing.

## Introduction

Wellbeing is a broad concept encompassing positive functioning and mental health, including factors such as life satisfaction and happiness [1–3]. Wellbeing is associated with many positive outcomes, including positive relationships, better physical health, and favourable work consequences [4]. Higher levels of wellbeing not only result from more positive outcomes, but also lead to them, so it is important for researchers to explore how to improve wellbeing. Previous research has shown that positive interventions can be effective [5,6]. Such interventions include gratitude activities [7–10], acts of kindness [11,12], and imagining one’s best possible self [8,13,14]. These wellbeing interventions can be cost effective and easy to implement, especially when conducted online.

Gratitude, which is the acknowledgment by an individual of the external sources of benefits received [15], has consistently been shown to relate to higher wellbeing (such as positive affect and life satisfaction) and lower depressive symptomology [16]. Due to this connection, several gratitude interventions have been developed that promote positive affect and other aspects of wellbeing. Gratitude intervention activities include creating gratitude lists, writing letters of gratitude, and expressing gratitude directly to loved ones via gratitude visits [8,10,17].

Prosocial behaviour is defined as actions with the intention of benefitting others. It is also associated with improvements in wellbeing and mental health [18,19], and even predicts academic achievement in children [20]. One way that researchers have studied prosocial behaviour has been to instruct participants to perform acts of kindness. Performing acts of kindness may improve wellbeing by providing people with a sense of control and optimism in their ability to help [12]. Furthermore, kindness to others may encourage socializing and bonding between people. Doing several acts of kindness in one day each week has been shown to cause increases in wellbeing (as well as peer acceptance) in children [11] and college students [12], as well as in the general population [21,22].

Although growing evidence suggests that wellbeing interventions can effectively improve wellbeing, less research has investigated individual differences in intervention response. What are the characteristics that determine why some people respond better to a wellbeing intervention than others [23,24]? One possibility is that wellbeing interventions will be most effective when the characteristics of the participant and the characteristics of the wellbeing tasks are optimally matched (i.e. person-activity fit) [24]. If researchers can identify these salient characteristics then interventions could be designed to be maximally beneficial for all, for example through personalisation of activities, timing, duration or support. Understanding individual differences in intervention response may also help to uncover the mechanisms that drive the intervention effect.

Several measures have been explored for their potential moderation effects in wellbeing interventions. These are personality [25], baseline positive affect [26,27], baseline gratitude [15,27], activity fit [5,14,28,29] and effort [9,10,13,29]. We can also draw suggestions of potentially important factors from the general wellbeing literature. Sex, socioeconomic status and season have all been associated with general wellbeing levels [30–35]. We decided to conduct exploratory analyses on these predictors. We noted that factors that predict variation in wellbeing itself may not have the same direction or magnitude of effect on variation in wellbeing intervention response.

In the current exploratory study, 15 potential moderators were selected based on current wellbeing intervention theory and suggestions from the general wellbeing literature. These included measures of demographics, seasonality, personality, baseline characteristics, activity fit, and effort. This is the first study to conduct a comprehensive exploration of potential moderators of wellbeing intervention response in an adolescent age group. Below we detail the previous research that led us to select these potential moderators. For our outcome measures, we operationalized wellbeing to include happiness and life satisfaction, and also measured symptoms of anxiety and depression, which we refer to as mental health.

### Demographics and seasonality as moderators

#### Sex

The literature on sex differences in wellbeing is mixed, depending on the particular aspect of wellbeing studied, but the overall trend is that women report lower levels of wellbeing than men [32]. Specifically, in adolescents (15 years old), females have been shown to report lower levels of self-esteem, happiness, and more past worries [30]. Females also tend to experience higher levels of depressive symptoms, with this discrepancy becoming apparent by the age of 15 [35]. It has also been suggested that different sets of genes may be influencing subjective wellbeing in men and women and driving the difference between them [36]. Previous research has therefore highlighted the importance of sex in explaining individual differences in wellbeing, but the mixed findings do not lead to a simple hypothesis about the role of sex in explaining how individuals will respond to a wellbeing intervention. An example can be seen in the depression literature—women tend to have higher levels of depression than men [37], but the same risk factors lead to depression in both men and women equally [38,39]. Thus a similar effect may be seen with wellbeing in that women may have lower baseline levels, but still respond equally to a wellbeing intervention as men. This drove us to explore the role of sex in explaining individual differences in response to our wellbeing intervention in teenagers.

#### Socioeconomic status

Some previous research has indicated a positive correlation between socio-economic status (SES) and levels of wellbeing [31–33], with measures of SES including indices of education, employment, income and social class. SES has also been related to the expression of gratitude [40], with a contrasting association, whereby pre-schoolers from families with low SES expressed gratitude through saying “thank you” more frequently than children from families with higher SES. Accordingly, low SES might predict greater improvements in wellbeing because (on average) this group, who tend to have lower wellbeing, may have more room for improvement. But the differing value of gratitude as a function of SES might mean that a gratitude intervention is differentially effective in low versus high SES groups, suggesting that expressing gratitude is a better “fit” for people from low-SES families. Previous research precludes a clear hypothesis about the direction of effects, so we will explore both possibilities in our analyses.

#### Seasonality

Finally, the limited literature on seasonality has shown that happiness tends to be higher in Spring than in Autumn (Fall) [34] and we wished to explore the effect of season on intervention response. Accordingly, in this study, we included the demographic factors of sex and SES, and the season in which the intervention was conducted as potential moderators.

### Personality as a moderator

The link between personality and wellbeing is well supported [41,42], including at the genetic level [43]. Higher levels of neuroticism have been linked to lower levels of happiness [43]. Furthermore, one study has provided preliminary evidence regarding the moderating role of personality for intervention response. In this study, participants were instructed to write and present letters of gratitude and to conduct daily reflections on three good things that they experienced. It was found that participants with higher levels of extraversion and openness experienced greater gains in happiness and greater decreases in depression in the gratitude condition [25]. In relation to a prosociality intervention, where personality as a moderator has not been explored, given the interpersonal nature of performing acts of kindness for others, this activity might also be a particularly good fit for people who are high in extraversion.

Another aspect of personality is sensation seeking, the degree to which an individual enjoys and searches for novel experiences. Sensation seeking has been shown to moderate the association between physical pleasure and life satisfaction in daily life [44]. Individuals high in sensation seeking may enjoy the novelty of completing intervention tasks and experience the greatest positive impact. Alternatively, over time, these intervention tasks may lose their novelty and end up having the smallest effect on this group compared with people low in sensation seeking.

### Baseline characteristics as moderators

Previous studies have produced inconclusive results when it comes to the moderating effects of baseline positive affect on wellbeing intervention response. In one study among youths asked to write and deliver a letter of gratitude, those with lower levels of positive affect experienced greater benefits at post-treatment and at follow-up, compared to those who only wrote about daily events [26]. In another study in which participants had to bring to mind someone or something for which they were grateful, no moderating effects of either positive or negative affect at baseline were found [27].

Similarly, given that the current study targeted gratitude and prosocial behaviour, baseline gratitude and prosociality might moderate the effects of the intervention. In the study conducted by Rash et al. [27], participants who were in the gratitude condition reported greater gains in life satisfaction when they had lower dispositional gratitude [15,27]. Based on this evidence obtained with gratitude-inducing interventions, we tested the moderating effect of baseline positive affect and baseline gratitude levels on intervention response. Given that our current intervention includes both gratitude (gratitude letters) and prosociality (kind acts) components, we also tested the moderation effects of baseline levels of prosociality.

### Activity measures as moderators

Participants may vary in how they carry out and react to intervention activities. Potential activity moderators include the degree of hedonic adaption, the fit of the person to the activity, whether the gratitude letters were shared with others, and the amount of effort put into completing the activities.

#### Hedonic adaptation

Hedonic adaptation refers to the adjustment to environmental changes that may initially influence wellbeing levels. For example, studies have shown that wellbeing shifts in response to life events, such as marriage, childbirth, and divorce, followed by a return to pre-event levels of wellbeing [45]. Adaptation may also occur as people engage in activities to improve their wellbeing, particularly when those activities are similar from week to week. Consequently, any increases or decreases in wellbeing may be transient. Indeed the literature suggests that hedonic adaptation is faster to positive, wellbeing-enhancing events, than to negative circumstances [46]. We explored individual differences in the rate at which people hedonically adjust to our wellbeing intervention and whether this has an effect on variation in how much people improve in their wellbeing.

#### Preferences and motivations

Supporting the notion of person-activity fit, participants with a strong preference for a wellbeing activity to which they are assigned tend to experience greater gains in their wellbeing [29,47]. Similarly, those who report the tasks as natural and enjoyable tend to reap the most benefits [28]. Initial motivation to perform a wellbeing-boosting task has also been shown to predict performance and outcomes of that task [14]. Participants who self-select into positive interventions have been found to experience greater gains than those who do not self-select [5]. We explored the moderating effects of perceiving a task as natural and enjoyable, and how much the individual was motivated to improve their wellbeing, in determining intervention response.

#### Gratitude letter sharing

Some prior gratitude interventions have required participants to deliver their letter to the addressee [e.g. 10,25] while others have not [8,13]. In addition to confounding the expression of gratitude with the social interaction inherent in gratitude delivery, we believe that requiring the participant to deliver their letter may not be suitable in an adolescent sample as it may lead to anxiety over how the addressee may receive the letter. Therefore, our gratitude intervention did not require the individuals to share their letter. The participants, however, were asked if they did share any letters, and we examined whether this led to a boost in wellbeing over and above the effect of merely writing gratitude letters.

#### Effort

Research supports the role of effort on wellbeing intervention response. Effort has been assessed through self-report [9] and ratings from independent coders [13]. Across these different modes of assessment, effort predicts greater increases in wellbeing in response to positive activities. Continued adherence to positive activities and continuation of the activities after the intervention period have also been shown to predict sustained wellbeing increase [10,29]. Effort and its related indices were also of interest in this current study.

### The current study

The aim of the current study is to better understand the moderators of wellbeing intervention response and advance this literature. We used data from the Twins Wellbeing Intervention Study [48], in which participants completed gratitude letters and performed acts of kindness for 3 weeks. It has already been shown that this intervention significantly improved wellbeing and decreased internalising symptomology [48]. The participants were 16 years old, a critical period of late adolescence. Most wellbeing interventions have focused on adult populations, and those that have examined child and adolescent samples usually explore this population over a large age range, with mean ages in early adolescence. Because childhood and adolescence are periods of rapid change, additional insights may be gained by focussing on specific age groups to accommodate the possibility of response heterogeneity across different ages. Our study focuses for the first time on a specifically narrow age range using a large sample size. In addition to investigating a rarely studied age group, this study also provides unique information on a large range of possible moderators of response to a wellbeing intervention.

## Method

### Participants

Participants were a subsample of the larger, population representative Twins Early Development Study [49]. Families were selected from TEDS to provide a subsample of same-sex twin pairs who were representative with respect to socioeconomic status, sex, and zygosity. Ethical approval was provided by the Institute of Psychiatry research ethics committee at King’s College London (Ref: PNM/10/11-16). Informed consent was obtained from both the twins themselves and from the twins’ parents/guardians on behalf of the twins, in written form. The sample comprised 932 individuals (55.6% females) with an average age of 16.55 (*SE* = 0.52) at time of consent. These individuals were nested in twin pairs. The data from 22 participants were excluded because they had experienced birth complications. Each participant completed the same tasks in the study. 884 participants provided the relevant baseline wellbeing and mental health responses for our analysis (subjective happiness, life satisfaction, anxiety and depression), and 805 (91%) continued to provide outcome responses at follow-up. These 884 participants were used in our analysis. For further information about the TWIST sample, please see [48].

### Study design

The participants took part in a 10-week within-person controlled online intervention study. This method is in line with the design of an n-of-1 study which has been shown to be feasible and useful in educational and clinical settings [50], and is a step towards personalising interventions [51]. Participants logged on each week to complete a range of assessments, as well as to receive instructions for their tasks. Baseline measures were collected in week 0, followed by 3 weeks of control tasks in the control phase, then 3 weeks of wellbeing tasks in the intervention phase. The participants then had a 3-week break before completing follow-up assessments in the follow-up phase. For the control tasks, participants were instructed to take note of three places they visited on one day of each week and were asked to write a detailed description of one room in their home each week. For the intervention tasks, the participants had to perform three acts of kindness on one day of each week and write a letter of gratitude to someone important in their lives each week. Outcome measures were assessed at milestone weeks 0 (baseline), 3 (end of the control phase), 6 (end of intervention phase) and 9 (end of follow-up phase). The study was split into two waves of participants, with 285 participants (32%) taking part in the study in Autumn 2012 and 598 participants (68%) taking part in the study in Spring 2013.

### Measures

#### Outcomes

Our outcome variables were wellbeing and mental health. Wellbeing was a standardized composite of responses for the 4-item Subjective Happiness Scale [52] and the 6-item Brief Multidimensional Student Life Satisfaction Scale [53]. Over all 4 data collection time points, these two questionnaires showed good internal consistency with Cronbach’s alphas ranging from 0.86 to 0.88 for SHS, and 0.85 to 0.88 for BMSLSS. Mental health was a standardised composite of the responses for the short (13-item) Moods and Feelings Questionnaire [54] and the 6-item State-Trait Anxiety Inventory [55]. These questionnaires, measuring symptoms of mental illness, were reverse scored so that a higher value of the composite indicated better mental health. Cronbach’s alphas for these two measures ranged from 0.90 to 0.91 for MFQ and 0.79 to 0.83 for STAI.

#### Moderators

Details of the moderators are shown in Table 1, including information on the number of items in each scale, item scoring, an example item, and the reference for the published scale. In total, we used 15 moderators. All measures demonstrated good internal consistency reliability in our sample apart from the personality subscales that showed low correlations. However, with only two items per construct, the low correlations were expected, reflecting that these two items try to eliminate item redundancy and minimise content overlap, at the expense of internal consistency [56].

**Table 1:** Potential moderators of intervention response.

Construct	Time of assessment	Measure Name	Number of items	A sample item	How items are scored	Higher score represents	Internal Consistency (Cronbach’s alpha)
*Demographic factors*							
Sex	First TEDS contact: when participants were 18 months old	n/a	n/a	n/a	Dummy coded	Male	n/a
					0: female		
					1: male		
Socioeconomic status	First TEDS contact: when participants were 18 months old	n/a	5	n/a	Composite of 5 derived variables relating to parent qualifications and employment, and mother's age at birth of first child	Higher SES	n/a
*Seasonality*							
Season	n/a	n/a	n/a	n/a	Dummy coded	Spring 2013 wave	n/a
					0: Autumn 2012 wave		
					1: Spring 2013 wave		
*Personality and Sensation seeking*							
Personality	Baseline (week 0)	10 item personality inventory [56]	10	"I see myself as: Extraverted, enthusiastic"	7 point scale from "disagree strongly" to "agree strongly"	Higher extraversion, agreeableness, conscientiousness, openness and lower neuroticism	Extraversion = 0.33
							Agreeableness = 0.13
							Conscientiousness = 0.33
							Neuroticism = 0.46
							Openness = 0.16
Sensation seeking	Baseline (week 0)	Brief Sensation Seeking Scale [57]	8	"I would like to explore strange places"	5 point scale from "strongly disagree" to "strongly agree"	Higher level of sensation seeking	0.78
*Baseline characteristics*							
Positive affect	Before control phase (week 0)	Positive subscale of Emotional Report [58]	4	"Please indicate the extent to which you have felt this way in the past week: Happy"	5 point scale from "not at all" to "most of the time"	Higher positive affect	Before control phase = 0.83
	Before intervention phase (week 3)						Before intervention phase = 0.88
Gratitude	Before intervention phase (week 3)	Gratitude Questionnaire [59]	6	"I have so much in life to be thankful for."	7 point scale from "disagree strongly" to "agree strongly"	Higher level of gratitude	0.81
Prosociality	Before intervention phase (week 3)	Prosocial subscale of Strengths and Difficulties Questionnaire [60]	5	"I try to be nice to other people"	3 point scale of "not true", " somewhat true" or "very true"	Higher prosociality	0.74
*Activity moderators*							
Hedonic adaptation	After control phase (week 3)	n/a	2	"To what extent did you get bored with describing a room?"[14]	7 point scale from "not at all bored" to "extremely bored"	Higher hedonic adjustment to control/intervention activities in each consecutive phase	n/a
	After intervention phase (week 6)						
Person activity fit	Baseline (week 0)	n/a	9	"How enjoyable would it be for you to write down your daily activities on a regular basis?" [24]	7 point scale from "not at all enjoyable/natural" to "extremely enjoyable/natural"	Higher fit to control/intervention tasks and more motivated to becoming happier	n/a
Shared gratitude letters	Follow-up (week 9)	n/a	1	"You wrote three letters of gratitude as part of the study. How many (if any) did you share with someone else?"	Dummy coded	1 or more gratitude letters shared	n/a
					0: no letters shared		
					1: 1 or more letters shared		
Self-reported effort	Control phase: week 1, 2, 3	n/a	1	"How much effort did you put into completing this week’s activities?"	7 point scale from "no effort at all" to "a great deal of effort"	More self-reported effort in control/intervention phase	n/a
	Intervention phase: week 4, 5, 6						
Task effort	Control phase: week 1, 2, 3	n/a	n/a	n/a	Composite of the average number of characters written (i.e. the length of the written response), and the amount of time spent on the written activity each week	More effort in control/intervention phase as indicated by their task response	n/a
	Intervention phase: week 4, 5, 6						
Continuation	Follow-up (week 9)	n/a	1	"Have you continued to do acts of kindness for people since the end of the study?"	Dummy coded	Continuation with wellbeing tasks into follow-up	n/a
					0: continued with wellbeing tasks		
					1: did not continue with wellbeing tasks		
Number of practical activities	Control phase: week 1, 2, 3	n/a	n/a	n/a	Count	Larger number of activities completed in control/intervention phase	n/a

Note. SES was also measured in a TEDS subsample when the twins were 16 years old, but none of the TWIST subsample participants were part of the subsample that provided this 16-year SES measure. The 16-year SES from the TEDS sample was strongly correlated with the 18-month SES (Pearson’s r = .70), suggesting the 18-month SES should be an adequate proxy for current SES in the TWIST participants.

### Statistical analysis

To correct for negative skew in the wellbeing and mental health outcome measures, a van der Waerden rank transformation was applied. Piecewise hierarchical linear mixed models were fitted to the data, predicting changes in wellbeing and mental health in the control phase, intervention phase, and follow-up phase. This model allows the fitting of within-individual repeated measures data in which outcome measures (level 1) are nested within participants (level 2) who are nested within families (level 3). In addition to fixed parameter estimates, which inform us about average changes in outcome over the three phases, random parameters are estimated that give indications of individual variability in this change.

First, a basic piecewise hierarchical linear mixed model was fitted with wellbeing as the outcome and no level 2 predictors, similar to analysis previously conducted [48]. This gave an indication of general change in outcome response due to the three phases (fixed effects), and the individual differences in intervention response (random effects). Next, the potential level 2 predictors of interest were explored. Empirical Bayes residuals for each participant’s individual slopes (individual change in outcome in each of the three phases) were obtained from the basic model. These were regressed on the potential predictors in a series of univariate regressions. The predictors which produced regression results with t-to-enter values of more than 1 were selected to be included into the final interaction model [61]. An interaction model was then fitted, with wellbeing as the outcome variable and including the potential level 2 predictors that were selected from the univariate regressions of the previous step. This model was used to reveal the significant moderators of outcome change. These steps were repeated using mental health as the outcome.

A large number of interaction effects were assessed for statistical significance in the hierarchical models to test for the potential moderation effects. To correct for multiple testing, a Bonferroni significance level was applied.

## Results

### Descriptive statistics

No mean differences emerged between those who provided outcome information at baseline and continued to provide information at follow-up compared to those who dropped out by follow-up in terms of SES, baseline wellbeing levels and baseline mental health levels (S1 Table). All potential predictors showed variance inflation factors (VIF) of less than three, indicating no problematic collinearity (S2 Table). The basic piecewise models for wellbeing (S3 Table) and mental health (S8 Table) response produced the same pattern of results as previously found in this sample [48]. We note that the results are not identical to the previous analysis on this sample because of differences in exclusion criteria between the two analyses, namely that we included number of activities completed as a potential moderator rather than as part of the exclusion criteria as in the previous analysis. We obtained empirical Bayes residuals for each participant’s individual slopes in each of the three phases, regressing these on each of our potential moderators to obtain t-to-enter statistics (S4 and S9 Tables). From this exploratory t-to-enter stage, 20 interaction effects were put into the final wellbeing model (Table 2 and S5 Table) and 26 interaction effects were put into the final mental health model (Table 3 and S10 Table).

**Table 2:** Moderator model for wellbeing response.

Fixed parameter (interaction effects)	Coefficient	SE	*p*-value
Period 1, Control Phase (β_1_)			
Effect of sex, γ_11_	8.07e-02	4.30e-02	0.06
Effect of year 1 SES, γ_12_	-8.28e-03	2.00e-02	0.68
Effect of extraversion, γ_13_	1.60e-02	1.52e-02	0.29
Effect of neuroticism, γ_14_	-1.35e-02	1.49e-02	0.37
Effect of sensation seeking, γ_15_	-3.09e-02	2.65e-02	0.24
Effect of hedonic adaptation to control tasks, γ_16_	-7.72e-04	1.35e-02	0.95
Effect of self-reported effort during control phase, γ_17_	6.76e-02	2.10e-02	1.31e-03**†
Effect of task effort in control phase, γ_18_	-3.29e-02	2.27e-02	0.15
Period 2, Intervention Phase (β_2_)			
Effect of sex, γ_21_	-3.98e-02	4.21e-02	0.35
Effect of study wave, γ_22_	3.03e-02	4.15e-02	0.46
Effect of agreeableness, γ_23_	1.36e-02	1.75e-02	0.44
Effect of positive affect before intervention phase, γ_24_	-9.92e-03	6.16e-03	0.11
Effect of gratitude before intervention phase, γ_25_	-3.47e-02	2.37e-02	0.14
Effect of prosociality before intervention phase, γ_26_	2.41e-02	1.07e-02	2.47e-02*
Effect of hedonic adaptation to wellbeing tasks, γ_27_	-1.69e-02	1.28e-02	0.19
Effect of fit to wellbeing tasks, γ_28_	-3.27e-03	1.82e-02	0.86
Effect of task effort in intervention phase, γ_29_	2.80e-02	2.08e-02	0.18
Period 3, Follow-up Phase (β_2_)			
Effect of sex, γ_31_	0.12	4.28e-02	5.10e-03**
Effect of year 1 SES, γ_32_	1.57e-02	1.88e-02	0.40
Effect of study season, γ_33_	-9.20e-02	4.60e-02	4.58e-02*
**Random effects**	**SD**		
Level 1:			
Level 1 error	0.11		
Level 2:			
Intercept	0.22		
Control phase	8.61e-02		
Intervention phase	0.11		
Follow-up phase	0.15		
Level 3:			
Intercept	0.50		
Control phase	0.46		
Intervention phase	0.46		
Follow-up phase	0.46		
AIC	3611.36		
BIC	3969.25		
logLike	-1744.68		

*p < .05.

**p < .01.

†*p*<2.50e-03 (Bonferroni).

*N* = 654 twins in 360 families, 2610 observations.

*Note*: This is a piecewise hierarchical linear mixed effects model for predicting changes in wellbeing and potential level 2 predictors of individual differences in response. The 3 levels of the model incorporate repeated measures nested in twins nested in families. This table only shows the interactions effects of the model, relevant for our analysis. For the full model, see S5 Table. Number of families, twins and observations used differ across our analysis as the multilevel model removes cases which have missing values in relevant predictors. The basic model was rerun using only compete cases to keep sample size the same as here (S6 Table) and comparable results were found. S7 Table shows the fit statistics comparing across models using only cases with complete data. S13 Table shows the complete interaction model for wellbeing response removing self-reported effort and task effort as predictors (to increase sample size).

**Table 3:** Moderator model for mental health response.

Fixed parameter (interaction effects)	Coefficient	SE	*p*-value
Period 1, Control Phase (β_1_)			
Effect of sex, γ_11_	9.73e-02	6.71e-02	0.15
Effect of year 1 SES, γ_12_	-7.02e-02	3.00e-02	1.95e-02*
Effect of study wave, γ_13_	8.54e-02	6.87e-02	0.21
Effect of extraversion, γ_14_	6.49e-03	2.25e-02	0.77
Effect of neuroticism, γ_15_	-3.61e-02	2.48e-02	0.15
Effect of initial positive affect before control phase, γ_16_	-3.57e-02	1.14e-02	1.80e-03**†
Effect of hedonic adaptation to control tasks, γ_17_	-5.01e-03	1.97e-02	0.80
Effect of self-reported effort in control tasks, γ_18_	9.77e-03	3.14e-02	0.76
Period 2, Intervention Phase (β_2_)			
Effect of sex, γ_21_	-0.12	6.18e-02	6.21–02
Effect of study wave, γ_22_	9.95e-02	6.20e-02	0.11
Effect of agreeableness, γ_23_	5.41e-02	2.37e-02	2.25e-02*
Effect of conscientiousness, γ_24_	1.28e-02	2.15e-02	0.55
Effect of neuroticism, γ_25_	-2.87e-02	2.02e-02	0.15
Effect of initial positive affect before intervention phase, γ_26_	-1.11e-02	8.74e-03	0.20
Effect of initial gratitude before intervention phase, γ_27_	-2.27e-02	3.30e-02	0.49
Effect of hedonic adaptation to wellbeing tasks, γ_28_	-2.57e-02	1.82e-02	0.16
Effect of fit to wellbeing tasks, γ_29_	1.82e-02	2.36e-02	0.44
Effect of sharing gratitude letters, γ_210_	-0.11	6.02e-02	8.11e-02
Effect of self-reported effort in intervention phase, γ_211_	8.04e-03	2.45e-02	0.74
Effect of task effort in in intervention phase, γ_212_	3.19e-02	2.84e-02	0.26
Period 3, Follow-up Phase (β_2_)			
Effect of sex, γ_31_	0.19	6.75e-02	4.74e-03**
Effect of year 1 SES, γ_32_	2.53e-02	3.04e-02	0.41
Effect of study wave, γ_33_	-0.13	7.25e-02	7.99e-02
Effect of conscientiousness, γ_34_	-2.63e-02	2.56e-02	0.30
Effect of motivation to becoming happier, γ_35_	9.88e-03	2.35e-02	0.67
Effect of sharing gratitude letters, γ_36_	-5.23e-02	7.34e-02	0.48
**Random Effects**	**SD**		
Level 1:			
Residual (e_i_)	0.23		
Level 2:			
Intercept	0.22		
Control phase	0.24		
Intervention phase	0.16		
Follow-up phase	0.30		
Level 3:			
(Intercept, U_0_)	0.55		
Control phase (U_1_)	0.63		
Intervention phase (U_2_)	0.59		
Follow-up phase (U_3_)	0.65		
AIC	5158.04		
BIC	5562.40		
logLik	-2510.02		

*p < .05.

**p < .01.

†*p*<1.92e-03 (Bonferroni).

N = 648 twins in 358 families, 2592 observations.

Note: This is a piecewise hierarchical linear mixed effects model for predicting changes in mental health and potential level 2 predictors of individual differences in response. The 3 levels of the model incorporate repeated measures nested in twins nested in families. This table only shows the interactions effects of the model, relevant for our analysis. For the full model, see S10 Table. Number of families, twins and observations used differ across our analysis as the multilevel model removes cases which have missing values in any of the relevant predictors. The basic model was rerun using only compete cases (S11 Table) and comparable results were found. S12 Table shows the fit statistics comparing across models using only cases with complete data. S14 Table shows the complete interaction model for mental health response removing self-reported effort and task effort as predictors (to increase sample size).

### Wellbeing

Table 2 shows the interaction effects we included within the full interaction model with wellbeing as the outcome (S5 Table shows the complete results from the model). The Bonferroni corrected α, to correct for multiple testing for all 20 interaction effects of interest, was 0.0025. Only self-reported effort during the control phase significantly explained individual differences in changes in wellbeing during the control phase, after Bonferroni correcting for multiple testing (γ = 0.07, SE = 0.02, p = 0.0013). This suggests that those who reported exerting more effort experienced greater increases in their wellbeing levels during the control phase. Some moderators were nominally significant as moderators of the intervention and follow-up phase at 0.05 and 0.01 alpha levels (see Table 2) but none reached Bonferroni significance in the these phases.

### Mental health

Table 3 shows the interaction effects included within the full interaction model with mental health as the outcome (S10 Table shows the complete results from the model). The Bonferroni corrected α, to correct for multiple testing for all 26 interaction effects of interest, was 0.0019. Only baseline positive affect during the control phase significantly explained individual differences in changes in mental health during the control phase, after Bonferroni correcting for multiple testing (γ = -0.04, SE = 0.01, p = 0.0018). This finding suggests that those with lower levels of baseline positive affect experienced greater improvements in mental health during the control phase. Again, some moderators were nominally significant as moderators of the intervention and follow-up phase at 0.05 and 0.01 alpha levels (see Table 3) but none reached Bonferroni significance in the these phases.

## Discussion

The literature is sparse on characteristics that predict individual differences in response to wellbeing interventions. This study provided the unique opportunity to study a large number of potential moderators on a large sample of adolescents within a specific age range. Due to the large number of interactions we examined, we corrected for multiple testing to reduce the chance of making Type 1 errors. Upon Bonferroni correlation, only two of our moderators were significant for change in wellbeing and mental health respectively, with effects seen only during the control phase of the study. We found that those who reported exerting more effort experienced greater increases in their wellbeing levels during the control phase, while those with lower levels of baseline positive affect experienced greater improvements in mental health during the control phase. Since participants were informed at the start of the study that they were taking part in an intervention designed to improve their wellbeing, self-reported effort and baseline positive affect may be proxy indicators of having a higher expectation of gaining positive results [13,62]. The reason why this is only important in the control phase may be that this expectancy is greatest at the start of the study and has a decreasing effect as the study progresses into the intervention and follow-up phases. In addition, this expectancy effect may cease to have an effect when the actual intended effect of the intervention comes into play (in the intervention phase). While on average, there was no significant change in wellbeing and mental health scores in the control phase, for a select number of individuals (those who exerted the most effort and have a lower baseline level of positive affect), the control tasks may in themselves be rewarding. Similar to mindfulness activities, these control tasks are reflective and encourage focus on simple activities, which a subset of people may benefit from. Furthermore, simply completing the task can be rewarding in itself by giving this subset of participants a sense of accomplishment.

None of the moderators we tested reached Bonferroni significance during the intervention phase and follow-up phase. This suggests that the wellbeing tasks had a pervasive positive effect on individuals regardless of sex, SES, season, personality, baseline characteristics or activity characteristics. Inequality in wellbeing is of increasing concern for government and policy makers [63,64]. Inequalities in wellbeing have previously been shown to be partly predicted by factors such as SES [32,33] and personality [41–43]. It is therefore interesting that these same factors are not a barrier for improvement during a wellbeing intervention. This suggests that these easy-to-implement tasks could be useful in improving the wellbeing and resilience of adolescents in a population-wide setting, without widening existing inequalities.

Previous between-group intervention studies have shown that effort, as rated by external judges, predicts an increase in wellbeing in the wellbeing group but not in the control group [13], and self-reported effort also predicts similar effects [9]. Between-group studies have produced mixed results for the importance of baseline positive affect as a moderator of intervention response [26,27]. Our finding that self-reported effort and baseline positive affect moderated responses in the control phase but not in the intervention phase is contrary to some of these previous findings. Interestingly, in their cross-cultural study, Layous and colleagues [9] found that self-reported effort was a more important moderator of intervention response in their U.S. sample compared to their South Korean sample. Thus, it could be cultural differences in our U.K. sample, in contrast to previous U.S. samples that are causing these differences in findings. Additionally, there is some evidence from the between-group studies of possible age-related differences in moderator effects [26,27] that could apply here to our 16-year-old sample, as different results have previously been found in adult samples compared to younger samples. Furthermore, it may be due to differences in the measurement of the moderator (e.g. self-report versus external raters), use of different outcome measures (e.g. positive affect and gratitude versus life satisfaction, happiness and internalising symptoms), the use of a between-group versus a within-group design, and length and span of the intervention (e.g. instructions to complete the gratitude task 5 times over 2 weeks versus once a week for 3 weeks). Further work is needed to reconcile these differences in findings for the impact of effort and baseline positive affect on intervention response. In addition, although it is very positive that this intervention was equally effective for all, there remains some variation in intervention response to be explained. This remaining variation is either random or moderated by other factors not considered here. A key future direction will be in identifying and testing other potential moderators.

### Study strengths and limitations

There are several strengths and limitations to our study. Below we consider the potential impact of aspects of our study design on the results, including the use of the within-individual design, using twins rather than singletons, cross-cultural differences, using a conservative Bonferroni correction, and other mechanisms that may confound the effect of the intervention on wellbeing.

This study was a within-participant study with the participants acting as their own controls. This is in line with the design of an n-of-1 study which recognises and objectively explores individual differences in intervention response. N-of-1 trials has been argued to be of immense utility in health and clinical research and should be demanding more attention [51]. The within-participant design allowed us to remove the error variance that exists in a between-subject design due to the possibility of sample differences between experimental conditions. Furthermore, we were able to increase the sample size and thus increase the power of the study; this is important in studies exploring moderators because interaction effects need more power to be detected than main effects. However, a limitation of the within-individual control design is that it cannot provide definitive evidence for the intervention causally increasing wellbeing—an external unmeasured factor occurring at the same time as the intervention could have caused the average increases in wellbeing and mental health (though this coincidence is unlikely).

Using twin pairs allowed for the novel examination of the importance of genetic and environmental influences on creating individual differences in intervention response [48]. One criticism of the twin design is that results are not generalizable to a singleton population. However, there is no reason why wellbeing and mental health would be different in twins versus singletons, or that the way in which these adolescents responded to the intervention was influenced by their family structure. Beyond early childhood, studies have shown twins to be no different to singletons in a variety of traits, such as psychopathology [65], personality [66], antisocial behaviour [67] and cognitive abilities [68]. We also corrected for the relatedness of the sample in all of our analyses.

The majority of previous wellbeing intervention studies have come from the U.S., using college-aged participants. In contrast, our study consisted of UK teenagers, an important but under-unexplored group in the wellbeing literature. Cultural differences may mean that different moderators are important to this UK adolescent sample in comparison to US adults. However, the smaller geographical and age range of our sample may have limited us in the amount of variation we were able to observe in the moderators.

While we were able to tackle a potential limitation of multiple testing by adjusting the alpha level using Bonferroni correction, this is a highly conservative method as it assumes that all tests are independent of each other, which in our analysis is not the case. However, given the size of the *p*-values, it is unlikely that less stringent corrections would have dramatically changed our conclusions. As an additional check, we conducted a power analysis for one of our potential moderators, agreeableness. We selected this one because it was first alphabetically in our list, and there was no reason to suspect that power would differ greatly across our different moderators. In this power analysis, using a simulation based approach, we found that using our sample size of 360 families (as used in our final moderation model), we have 80% power to detect an effect size as small as 0.063 for the interaction effect of agreeableness during the intervention phase on our wellbeing outcome (S1 Fig). Given that we have power to detect such small interaction effects, we can confidently rule out any very large moderation effects of clinical significance for intervention response, and supports our conclusions.

Finally, some increases in wellbeing in the participants may be due to the confounding effect of simply completing questionnaires on wellbeing. Completing wellbeing questionnaires may increase one’s emotional intelligence and this has been shown to be positively associated with wellbeing [69]. However, if this were the case, participants would have also experienced significant increases in wellbeing in the control phase.

### Conclusions

We were interested in understanding why some people respond more positively to wellbeing interventions than others. We tested a large number of potential moderators of intervention response to explore this question in an adolescent UK sample. Self-reported effort and baseline positive affect were Bonferroni significant moderators for changes in wellbeing and mental health, respectively, in the control phase. We speculate that these could be proxy indicators for levels of expectation in the positive intervention effect. No Bonferroni significant moderation effects were found during the intervention and follow-up phases. This is especially interesting as it suggests that the factors that are normally predictive of wellbeing inequality, such as sex, SES and personality, are not barriers to enabling people to positively respond to a wellbeing intervention such as ours. We believe this adds strength to the case for using a relatively low-cost and easy-to-implement intervention such as this to improve adolescent wellbeing at a population-wide level.

## Supporting information

S1 Table Difference between drop outs and non dropouts. (DOCX)

S2 Table Variance inflation factors of potential predictors. (DOCX)

S3 Table Basic model for wellbeing response. (DOCX)

S4 Table Exploratory t-to-enter statistics for potential level 2 predictors of wellbeing change. (DOCX)

S5 Table Complete results for interaction model for wellbeing response. (DOCX)

S6 Table Basic model for wellbeing response using cases with complete predictor information. (DOCX)

S7 Table Fit statistics for wellbeing outcome models. (DOCX)

S8 Table Basic model for mental health response. (DOCX)

S9 Table Exploratory t-to-enter statistics for potential level 2 predictors of mental health change. (DOCX)

S10 Table Complete results for interaction model for mental health response. (DOCX)

S11 Table Basic model for mental health response using cases with complete predictor information. (DOCX)

S12 Table Fit statistics for mental health outcome models. (DOCX)

S13 Table Complete results for interaction model for wellbeing response, not including effort measures as predictors. (DOCX)

S14 Table Complete results for interaction model for mental health response, not including effort measures as predictors. (DOCX)

S1 Fig Power curve of interaction effect of agreeableness during the intervention phase for wellbeing outcome. (DOCX)
