# Supplementary material for: Moderators of wellbeing interventions: Why do some people respond more positively than others?
Source: PLoS One. 2017 Nov 6;12(11):e0187601. doi: 10.1371/journal.pone.0187601 (PMC5673222; doi:10.1371/journal.pone.0187601)
Supplement: S1 Table — (DOCX) [file pone.0187601.s001.docx]

S1 Table. Difference between drop outs and non dropouts

|  | **Non dropout, N=807**  **Mean (SD)** | **Dropout, N=77**  **Mean (SD)** | **t-value (p-value)** |
| --- | --- | --- | --- |
| Socioeconomic status | 0.31 (0.96) | 0.10 (0.94) | 1.81 (0.07) |
| Baseline wellbeing | 0.00 (0.92) | 0.01 (0.93) | -0.12 (0.91) |
| Baseline mental health | 0.00 (0.89) | -0.07 (0.81) | 0.70 (0.49) |

*Note*. Socioeconomic status (SES) was assessed at TEDS first contact, when participants were 18 months old. It is composite of 5 derived variables relating to parent qualifications and employment, and mother’s age at birth of first child.

Wellbeing is a standardised composite of responses for the Subjective Happiness Scale (Lyubomirsky & Lepper, 1999) and the Brief Multidimensional Student Life Satisfaction Scale (Seligson, Huebner & Valois, 2003). Baseline wellbeing was measured in week 0, at the start of the study.

Mental health was a standardised composite of the responses for the short Moods and Feelings Questionnaire (Angold et al., 1995) and the State-Trait Anxiety Inventory (Spielberger & Gorsuch, 1983). It was reversed scaled so that a higher value represents better mental health. Baseline mental health was also measured at week 0, at the start of the study.

T-tests were conducted to compare SES, baseline wellbeing and baseline mental health between those who provided data at baseline and continued until followed up and those who provided data at baseline but had dropped out by follow-up, showing no mean differences.
