# Supplementary material for: Moderators of wellbeing interventions: Why do some people respond more positively than others?
Source: PLoS One. 2017 Nov 6;12(11):e0187601. doi: 10.1371/journal.pone.0187601 (PMC5673222; doi:10.1371/journal.pone.0187601)
Supplement: S2 Table — (DOCX) [file pone.0187601.s002.docx]

S2 Table. Variance inflation factors of potential predictors

|  | **VIF** |
| --- | --- |
| Sex | 1.33 |
| SES | 1.20 |
| Study wave | 1.13 |
| Extraversion | 1.48 |
| Agreeableness | 1.40 |
| Conscientiousness | 1.36 |
| Neuroticism | 1.55 |
| Openness | 1.44 |
| Sensation seeking | 1.29 |
| Positive affect week 0 | 1.73 |
| Positive affect week 3 | 1.69 |
| Gratitude week 3 | 1.99 |
| Prosociality week 3 | 1.60 |
| Hedonic adaptation (control tasks) | 1.42 |
| Hedonic adaptation (intervention tasks) | 1.58 |
| Fit to control tasks | 1.54 |
| Fit to intervention tasks | 2.05 |
| Motivation to becoming happier | 1.96 |
| Shared gratitude letters | 1.15 |
| Self-reported effort (control tasks) | 2.01 |
| Self-reported effort (intervention tasks) | 2.24 |
| Task effort (control tasks) | 1.66 |
| Task effort (intervention tasks) | 1.74 |
| Continuation of gratitude letters | 1.21 |
| Continuation of acts of kindness | 1.38 |
| Number of activities completed in control phase | 1.36 |
| Number of activities completed in intervention phase | 1.55 |

*Note.* All variance inflation factors are less than 3 showing that the potential predictors included in the model show no problematic collinearity.
