# Supplementary material for: Moderators of wellbeing interventions: Why do some people respond more positively than others?
Source: PLoS One. 2017 Nov 6;12(11):e0187601. doi: 10.1371/journal.pone.0187601 (PMC5673222; doi:10.1371/journal.pone.0187601)
Supplement: S3 Table — (DOCX) [file pone.0187601.s003.docx]

S3 Table. Basic model for wellbeing response

| **Fixed effect** | **Coefficient (SE)** | ***p*-value** |
| --- | --- | --- |
| (Intercept, β_0_) |  |  |
| γ_00_ | -6.29e-02 (3.73e-02) | 9.21e-02 |
| Control phase (β_1_) |  |  |
| γ_10_ | -4.77e-03 (1.84e-02) | 0.80 |
| Intervention phase (β_2_) |  |  |
| γ_20_ | 9.85e-02 (1.84e-02) | 9.42e-08† |
| Follow-up phase (β_3_) |  |  |
| γ_30_ | 4.14e-02 (1.88e-02) | 2.82e-02* |
| **Random parameter** | **SD** | |
| Level 1: |  |  |
| Residual error (e_i_) | 0.15 |  |
| Level 2: |  |  |
| Intercept | 0.61 |  |
| Control phase | 0.13 |  |
| Intervention phase | 0.07 |  |
| Follow-up phase | 0.13 |  |
| Level 3: |  |  |
| Intercept (U_0_) | 0.68 |  |
| Control phase (U_1_) | 0.45 |  |
| Intervention phase (U_2_) | 0.46 |  |
| Follow-up phase (U_3_) | 0.44 |  |
| AIC | 5570.10 |  |
| BIC | 5722.62 |  |
| logLik | -2760.05 |  |

*p<.05, **p<.01, ***p<.001, †p<0.0125 (Bonferroni)

N= 884 twins in 452 families, 3298 observations

*Note*. Basic piecewise hierarchical linear mixed model showing significant improvement in wellbeing during the intervention phase. 3 levels incorporating repeated measures nested in twins nested in families.
