# Supplementary material for: Moderators of wellbeing interventions: Why do some people respond more positively than others?
Source: PLoS One. 2017 Nov 6;12(11):e0187601. doi: 10.1371/journal.pone.0187601 (PMC5673222; doi:10.1371/journal.pone.0187601)
Supplement: S4 Table — (DOCX) [file pone.0187601.s004.docx]

S4 Table. Exploratory t-to-enter statistics for potential level 2 predictors of wellbeing change

| **Potential Level 2 Predictors (moderators)** | **Level 1 Coefficient (β)** | | |
| --- | --- | --- | --- |
|  | **Control phase: β_1_** | **Intervention phase: β_2_** | **Follow-up phase, β_3_** |
| sex | **1.64** | **-1.74** | **2.53** |
| SES | **-1.46** | 0.55 | **1.00** |
| study wave | -0.49 | **1.57** | **-2.28** |
| Extraversion | **1.86** | 0.11 | -0.15 |
| Agreeableness | 0.38 | **1.02** | 0.61 |
| Conscientiousness | 0.41 | -0.29 | 0.24 |
| Neuroticism | **1.64** | -2.79 | 0.34 |
| Openness | 0.52 | -0.60 | 0.94 |
| Sensation Seeking | **-1.88** | 0.54 | 0.94 |
| Positive Affect Week 0 | -0.67 |  |  |
| Positive Affect Week 3 |  | **-3.30** |  |
| Gratitude week 3 |  | **-1.07** |  |
| Prosociality week 3 |  | **1.60** |  |
| Hedonic Adaptation (Control Tasks) | **-1.93** |  |  |
| Hedonic Adaptation (Intervention Tasks) |  | **-1.79** |  |
| Fit to control tasks | -0.14 |  |  |
| Fit to intervention tasks |  | **2.24** |  |
| Motivation to becoming happier | 0.67 | 0.51 | -0.45 |
| Shared gratitude letter |  | -0.15 | 0.15 |
| Self Reported Effort (Control Tasks) | **4.10** |  |  |
| Self Reported Effort (Intervention Tasks) |  | 0.82 |  |
| Task Effort (Control Tasks) | **-1.37** |  |  |
| Task Effort (Intervention Tasks) |  | **1.99** |  |
| Continuation of gratitude letters |  |  | -0.53 |
| Continuation of acts of kindness |  |  | -0.49 |
| Activities completed (Control phase) | 0.31 |  |  |
| Activities completed (Intervention phase) |  | 0.67 |  |

*Note*. Empirical Bayes residuals from the basic model were regressed on individual predictors (univariate regression). Numbers in bold indicate t-values>1, used as a criteria for inclusion in the interaction model, showing 20 interaction effects should be added (Raudenbush & Bryk, 2002).
