# Supplementary material for: Moderators of wellbeing interventions: Why do some people respond more positively than others?
Source: PLoS One. 2017 Nov 6;12(11):e0187601. doi: 10.1371/journal.pone.0187601 (PMC5673222; doi:10.1371/journal.pone.0187601)
Supplement: S5 Table — (DOCX) [file pone.0187601.s005.docx]

S5 Table. Complete results for interaction model for wellbeing response

| **Fixed parameter** | **Coefficient** | **SE** | ***p*-value** |  |
| --- | --- | --- | --- | --- |
| Intercept (β_0_) |  |  |  |  |
| γ_00_ | -4.32 | 0.24 | 3.50e-66 |  |
| Period 1, Control Phase (β_1_) |  |  |  |  |
| γ_10_ | -0.26 | 0.16 | 0.11 |  |
| Period 2, Intervention Phase (β_2_) |  |  |  |  |
| γ_20_ | 0.22 | 0.14 | 0.13 |  |
| Period 3, Follow-up Phase (β_2_) |  |  |  |  |
| γ_30_ | 4.36e-02 | 4.10e-02 | 0.29 |  |
| Main effect of sex | -5.91e-02 | 5.30e-02 | 0.27 |  |
| Main effect of SES | -4.11e-02 | 2.56e-02 | 0.11 |  |
| Main effect of study season | 1.12e-02 | 4.82e-02 | 0.82 |  |
| Main effect of extraversion | 9.21e-02 | 1.89e-02 | 1.75e-06 |  |
| Main effect of agreeableness | 5.48e-02 | 2.19e-02 | 1.28e-02 |  |
| Main effect of neuroticism | 0.22 | 1.87e-02 | 1.17e-25 |  |
| Main effect of sensation seeking | -7.39e-02 | 3.27e-02 | 2.46e-02 |  |
| Main effect of positive affect before intervention phase | 9.41e-02 | 7.84e-03 | 4.64e-27 |  |
| Main effect of gratitude before intervention phase | 0.29 | 3.06e-02 | 4.34e-19 |  |
| Main effect of prosociality before intervention phase | 1.15e-02 | 1.35e-02 | 0.39 |  |
| Main effect of hedonic adaptation to control tasks | -5.12e-02 | 1.72e-02 | 3.11e-03 |  |
| Main effect of hedonic adaption to wellbeing tasks | 3.34e-02 | 1.68e-02 | 4.83e-02 |  |
| Main effect of fit to wellbeing tasks | 5.64e-02 | 2.30e-02 | 1.49e-02 |  |
| Main effect of self-reported effort during the control phase | -4.27e-02 | 2.64e-02 | 0.11 |  |
| Main effect of task effort during the control phase | 1.55e-02 | 3.08e-02 | 0.62 |  |
| Main effect of task effort during the intervention phase | -3.71e-02 | 2.98e-02 | 0.22 |  |
| *Control phase interaction effects:* |  |  |  |  |
| Interaction effect of sex in control phase | 8.07e-02 | 4.30e-02 | 0.06 |  |
| Interaction effect of year 1 SES in control phase | -8.28e-03 | 2.00e-02 | 0.68 |  |
| Interaction effect of extraversion in control phase | 1.60e-02 | 1.52e-02 | 0.29 |  |
| Interaction effect of neuroticism in control phase | -1.35e-02 | 1.49e-02 | 0.37 |  |
| Interaction effect of sensation seeking in control phase | -3.09e-02 | 2.65e-02 | 0.24 |  |
| Interaction effect of hedonic adaptation to control tasks | -7.72e-04 | 1.35e-02 | 0.95 |  |
| Interaction effect of self-reported effort during control phase | 6.76e-02 | 2.10e-02 | 1.31e-03 |  |
| Interaction effect of task effort in control phase | -3.29e-02 | 2.27e-02 | 0.15 |  |
| *Intervention phase interaction effects:* |  |  |  |  |
| Interaction effect of sex in intervention phase | -3.98e-02 | 4.21e-02 | 0.35 |  |
| Interaction effect of study wave in intervention phase | 3.03e-02 | 4.15e-02 | 0.46 |  |
| Interaction effect of agreeableness in intervention phase | 1.36e-02 | 1.75e-02 | 0.44 |  |
| Interaction effect of positive affect before intervention phase | -9.92e-03 | 6.16e-03 | 0.11 |  |
| Interaction effect of gratitude before intervention phase | -3.47e-02 | 2.37e-02 | 0.14 |  |
| Interaction effect of prosociality before intervention phase | 2.41e-02 | 1.07e-02 | 2.47e-02 |  |
| Interaction effect of hedonic adaptation to wellbeing tasks | -1.69e-02 | 1.28e-02 | 0.19 |  |
| Interaction effect of fit to wellbeing tasks | -3.27e-03 | 1.82e-02 | 0.86 |  |
| Interaction effect of task effort in intervention phase | 2.80e-02 | 2.08e-02 | 0.18 |  |
| *Follow-up phase interaction effects:* |  |  |  |  |
| Interaction effect of sex in follow-up phase | 0.12 | 4.28e-02 | 5.10e-03 |  |
| Interaction effect of year 1 SES in follow-up phase | 1.57e-02 | 1.88e-02 | 0.40 |  |
| Interaction effect of study season in follow-up phase | -9.20e-02 | 4.60e-02 | 4.58e-02 |  |
| **Random effects** | **SD** | | | |
| Level 1: |  | | | |
| Level 1 error | 0.11 | | | |
| Level 2: |  | | | |
| Intercept | 0.22 | | | |
| Control phase | 8.61e-02 | | | |
| Intervention phase | 0.11 | | | |
| Follow-up phase | 0.15 | | | |
| Level 3: |  | | | |
| Intercept | 0.50 | | | |
| Control phase | 0.46 | | | |
| Intervention phase | 0.46 | | | |
| Follow-up phase | 0.46 | | | |
| AIC | 3611.36 | | | |
| BIC | 3969.25 | | | |
| logLike | -1744.68 | | | |

*N*= 654 twins in 360 families, 2610 observations

*Note*. This is a piecewise hierarchical linear mixed effects model for predicting changes in wellbeing and potential level 2 predictors of individual differences in response. Results show coefficients for all main and interaction effects. The 3 levels of the model incorporate repeated measures nested in twins nested in families.

There are fewer observations used in this model compared to the basic model. This is because multilevel modelling cannot account for missing values in predictor variables so the analysis has excluded cases that have missing values in any of the relevant predictors.

The basic model was rerun with the same participants as used in for the interaction model (S6 Table). Results from basic model with fewer observations were comparable with the original basic model.
