# Supplementary material for: Moderators of wellbeing interventions: Why do some people respond more positively than others?
Source: PLoS One. 2017 Nov 6;12(11):e0187601. doi: 10.1371/journal.pone.0187601 (PMC5673222; doi:10.1371/journal.pone.0187601)
Supplement: S6 Table — (DOCX) [file pone.0187601.s006.docx]

S6 Table. Basic model for wellbeing response using cases with complete predictor information

| **Fixed effect** | **Coefficient (SE)** | ***p*-value** |
| --- | --- | --- |
| (Intercept, β_0_) |  |  |
| γ_00_ | -6.21e-02 (4.10e-02) | 0.13 |
| Control phase (β_1_) |  |  |
| γ_10_ | 1.87e-03 (2.01e-02) | 0.93 |
| Intervention phase (β_2_) |  |  |
| γ_20_ | 0.10 (2.00e-02) | 4.14e-07† |
| Follow-up phase (β_3_) |  |  |
| γ_30_ | 3.36e-02 (2.12e-02) | 0.11 |
| **Random parameter** | **SD** | |
| Level 1: |  |  |
| Residual error (e_i_) | 0.15 |  |
| Level 2: |  |  |
| Intercept | 0.56 |  |
| Control phase | 0.11 |  |
| Intervention phase | 0.11 |  |
| Follow-up phase | 0.16 |  |
| Level 3: |  |  |
| Intercept (U_0_) | 0.69 |  |
| Control phase (U_1_) | 0.44 |  |
| Intervention phase (U_2_) | 0.44 |  |
| Follow-up phase (U_3_) | 0.44 |  |
| AIC | 4315.29 |  |
| BIC | 4461.97 |  |
| logLik | -2132.645 |  |

*p<.05, **p<.01, ***p<.001, †p<0.0125 (Bonferroni)

N= 654 twins in 360 families, 2610 observations

*Note*. Basic piecewise hierarchical linear mixed model predicting changes in wellbeing. 3 levels incorporating repeated measures nested in twins nested in families. Individuals who had missing values for any of the predictor variables added into the interaction model as displayed in S5 Table were excluded to check the effect of discrepancies in observations used. Comparing this current results table with the basic model results as displayed in S3 Table, results were very similar, with a significant improvement in wellbeing during the intervention phase.
