# Supplementary material for: Moderators of wellbeing interventions: Why do some people respond more positively than others?
Source: PLoS One. 2017 Nov 6;12(11):e0187601. doi: 10.1371/journal.pone.0187601 (PMC5673222; doi:10.1371/journal.pone.0187601)
Supplement: S7 Table — (DOCX) [file pone.0187601.s007.docx]

S7 Table. Fit statistics for wellbeing outcome models

| Model | df | AIC | BIC | logLik | Test | L.Ratio | p-value |
| --- | --- | --- | --- | --- | --- | --- | --- |
| 1. Intercept | 2 | 7261.60 | 7273.33 | -3628.80 |  | NA | NA |
| 1. Random Intercept | 3 | 4482.58 | 4500.18 | -2238.29 | 1 vs 2 | 2781.02 | 0 |
| 1. Random Intercept, 3 levels – repeated measures nested in twins nested in families | 4 | 4435.75 | 4459.22 | -2213.88 | 2 vs 3 | 48.83 | 2.79e-12 |
| 1. Random intercept and fixed slope predicted by time, 3 levels | 5 | 4379.65 | 4408.99 | -2184.83 | 3 vs 4 | 58.10 | 2.50e-14 |
| 1. Random intercept and 3 slopes (piecewise) predicted by 3 time phases, 3 levels | 7 | 4376.28 | 4417.35 | -2181.14 | 4 vs 5 | 7.37 | 2.51e-02 |
| 1. Random intercept and 3 random slopes predicted by 3 time phases, 3 levels | 25 | 4315.29 | 4461.97 | -2132.65 | 5 vs 6 | 96.99 | 7.84e-13 |
| 1. Interaction model: individual slopes predicted by potential moderators, 3 levels | 61 | 3611.36 | 3969.25 | -1744.68 | 6 vs 7 | 775.93 | 0 |

*N=* 654 twins in 360 families, 2610 observations for all models.

*Note.* Table comparing the fit statistics of the fitted models for wellbeing as an outcome of intervention response. Models are built up from a simple intercept model to a full 3 level interaction model. Only cases that have complete data for all predictors used in the final interaction model are used in all models. Results show that each model is a significantly better fit for the data than the previous.
