# Supplementary material for: Moderators of wellbeing interventions: Why do some people respond more positively than others?
Source: PLoS One. 2017 Nov 6;12(11):e0187601. doi: 10.1371/journal.pone.0187601 (PMC5673222; doi:10.1371/journal.pone.0187601)
Supplement: S8 Table — (DOCX) [file pone.0187601.s008.docx]

S8 Table. Basic model for mental health response

| **Fixed effect** | **Coefficient (SE)** | ***p*-value** |
| --- | --- | --- |
| (Intercept, β_0_) |  |  |
| γ_00_ | -7.46e-02 (3.56e-02) | 3.60e-02* |
| Control phase (β_1_) |  |  |
| γ_10_ | 5.47e-03 (2.75e-02) | 0.84 |
| Intervention phase (β_2_) |  |  |
| γ_20_ | 8.59e-02 (2.65e-02) | 1.12e-03† |
| Follow-up phase (β_3_) |  |  |
| γ_30_ | 5.62e-02 (3.00e-02) | 6.14e-02 |
| **Random parameter** | **SD** | |
| Level 1: |  |  |
| Residual error (e_i_) | 0.28 |  |
| Level 2: |  |  |
| Intercept | 0.51 |  |
| Control tasks phase | 0.25 |  |
| Wellbeing tasks phase | 0.19 |  |
| Follow-up phase | 0.31 |  |
| Level 3: |  |  |
| Intercept (U_0_) | 0.72 |  |
| Control tasks phase (U_1_) | 0.59 |  |
| Wellbeing tasks phase (U_2_) | 0.57 |  |
| Follow-up phase (U_3_) | 0.61 |  |
| AIC | 7285.30 |  |
| BIC | 7437.83 |  |
| logLik | -3617.651 |  |

*p<.05, **p<.01, ***p<.001, †p<0.0125 (Bonferroni)

N= 884 twins in 452 families, 3298 observations

*Note*. Basic piecewise hierarchical linear mixed model showing significant improvement in mental health during the intervention phase. 3 levels incorporating repeated measures nested in twins nested in families.
