# Supplementary material for: Moderators of wellbeing interventions: Why do some people respond more positively than others?
Source: PLoS One. 2017 Nov 6;12(11):e0187601. doi: 10.1371/journal.pone.0187601 (PMC5673222; doi:10.1371/journal.pone.0187601)
Supplement: S9 Table — (DOCX) [file pone.0187601.s009.docx]

S9 Table. Exploratory t-to-enter statistics for potential level 2 predictors of mental health change

| **Potential Level 2 Predictors (moderators)** | **Level 1 Coefficient (β)** | | |
| --- | --- | --- | --- |
|  | **Control phase: β_1_** | **Intervention phase: β_2_** | **Follow-up phase, β_3_** |
| sex | **2.03** | **-4.15** | **3.08** |
| ASES | **-2.20** | 0.57 | **1.01** |
| study wave | **1.92** | **1.32** | **-1.28** |
| Extraversion | **-1.28** | 0.85 | -0.86 |
| Agreeableness | 0.46 | **2.19** | -0.75 |
| Conscientiousness | -0.53 | **1.44** | **-1.54** |
| Neuroticism | **-1.26** | **-3.23** | 0.05 |
| Openness | -0.21 | 0.31 | 0.78 |
| Sensation Seeking | -0.55 | -0.05 | 0.08 |
| Positive Affect Week 0 | **-2.55** |  |  |
| Positive Affect Week 3 |  | **-4.85** |  |
| Gratitude week 3 |  | **-1.07** |  |
| Prosociality week 3 |  | 0.22 |  |
| Hedonic Adaptation (Control) | **-2.97** |  |  |
| Hedonic Adaptation (Intervention) |  | **-2.01** |  |
| Fit to control tasks | 0.60 |  |  |
| Fit to intervention tasks |  | **1.54** |  |
| Motivation to becoming happier | -0.38 | 0.21 | **-1.11** |
| Shared gratitude letter |  | **-2.18** | **-1.50** |
| Self Reported Effort (Control) | **1.60** |  |  |
| Self Reported Effort (Intervention) |  | **1.11** |  |
| Task Effort (Control) | -0.23 |  |  |
| Task Effort (Intervention) |  | **2.52** |  |
| Continuation of gratitude letters |  |  | 0.42 |
| Continuation of acts of kindness |  |  | 0.42 |
| Activities completed (Control) | -0.68 |  |  |
| Activities completed (Intervention) |  | 0.72 |  |

*Note*. Empirical Bayes residuals from the basic model were regressed on individual predictors (univariate). Numbers in bold indicate t-values>1, used as a criteria for inclusion in the interaction model, showing 26 interaction effects should be added (Raudenbush & Bryk, 2002).
