# Supplementary material for: Moderators of wellbeing interventions: Why do some people respond more positively than others?
Source: PLoS One. 2017 Nov 6;12(11):e0187601. doi: 10.1371/journal.pone.0187601 (PMC5673222; doi:10.1371/journal.pone.0187601)
Supplement: S10 Table — (DOCX) [file pone.0187601.s010.docx]

S10 Table. Complete results for interaction model for mental health response

| **Fixed parameter** | **Coefficient** | **SE** | ***p*-value** |  |
| --- | --- | --- | --- | --- |
| Intercept (β_0_) |  |  |  |  |
| γ_00_ | -3.60 | 0.27 | 1.46e-39 |  |
| Period 1, Control Phase (β_1_) |  |  |  |  |
| γ_10_ | 0.46 | 0.25 | 6.41e-02 |  |
| Period 2, Intervention Phase (β_2_) |  |  |  |  |
| γ_20_ | 0.10 | 0.24 | 0.67 |  |
| Period 3, Follow-up Phase (β_2_) |  |  |  |  |
| γ_30_ | 0.15 | 0.17 | 0.39 |  |
| Main effect of sex | 2.55e-02 | 5.92e-02 | 0.67 |  |
| Main effect of SES | -1.52e-02 | 2.86e-02 | 0.59 |  |
| Main effect of study season | -4.35e-02 | 5.91e-02 | 0.46 |  |
| Main effect of extraversion | -2.12e-02 | 2.18e-02 | 0.33 |  |
| Main effect of agreeableness | -4.51e-02 | 2.44e-02 | 6.58e-02 |  |
| Main effect of conscientiousness | 5.13e-02 | 1.91e-02 | 7.51e-03 |  |
| Main effect of neuroticism | 0.27 | 2.20e-02 | 3.81e-27 |  |
| Main effect of positive affect before control phase | 7.87e-02 | 1.19e-02 | 2.22e-10 |  |
| Main effect of positive affect before intervention phase | 5.33e-02 | 9.39e-03 | 3.52e-08 |  |
| Main effect of gratitude before intervention phase | 0.24 | 3.44e-02 | 3.21e-11 |  |
| Main effect of hedonic adaptation to control tasks | -4.01e-02 | 1.96e-02 | 4.22e-02 |  |
| Main effect of hedonic adaption to wellbeing tasks | 5.83e-04 | 1.94e-02 | 0.98 |  |
| Main effect of fit to wellbeing tasks | -4.49e-02 | 2.50e-02 | 7.38e-02 |  |
| Main effect of motivation to becoming happier | -6.49e-03 | 2.21e-02 | 0.77 |  |
| Main effect of sharing letter | -6.56e-02 | 5.28e-02 | 0.21 |  |
| Main effect of self-reported effort during the control phase | 2.03e-02 | 3.35e-02 | 0.55 |  |
| Main effect of self-reported effort during the intervention phase | -3.63e-02 | 2.80e-02 | 0.20 |  |
| Main effect of task effort during the intervention phase | -0.10 | 2.93e-02 | 1.11e-03 |  |
| *Control phase intervention effects:* |  |  |  |  |
| Interaction effect of sex in control phase | 9.73e-02 | 6.71e-02 | 0.15 |  |
| Interaction effect of year 1 SES in control phase | -7.02e-02 | 3.00e-02 | 1.95e-02 |  |
| Interaction effect of study wave in control phase | 8.54e-02 | 6.87e-02 | 0.21 |  |
| Interaction effect of extraversion in control phase | 6.49e-03 | 2.25e-02 | 0.77 |  |
| Interaction effect of neuroticism in control phase | -3.61e-02 | 2.48e-02 | 0.15 |  |
| Interaction effect of initial positive affect before control phase | -3.57e-02 | 1.14e-02 | 1.80e-03 |  |
| Interaction effect of hedonic adaptation to control tasks | -5.01e-03 | 1.97e-02 | 0.80 |  |
| Interaction effect of self-reported effort in control tasks | 9.77e-03 | 3.14e-02 | 0.76 |  |
| *Intervention phase intervention effects:* |  |  |  |  |
| Interaction effect of sex in intervention phase | -0.12 | 6.18e-02 | 6.21-02 |  |
| Interaction effect of study wave in intervention phase | 9.95e-02 | 6.20e-02 | 0.11 |  |
| Interaction effect of agreeableness in intervention phase | 5.41e-02 | 2.37e-02 | 2.25e-02 |  |
| Interaction effect of conscientiousness in intervention phase | 1.28e-02 | 2.15e-02 | 0.55 |  |
| Interaction effect of neuroticism in intervention phase | -2.87e-02 | 2.02e-02 | 0.15 |  |
| Interaction effect of initial positive affect before intervention phase | -1.11e-02 | 8.74e-03 | 0.20 |  |
| Interaction effect of initial gratitude before intervention phase | -2.27e-02 | 3.30e-02 | 0.49 |  |
| Interaction effect of hedonic adaptation to wellbeing tasks | -2.57e-02 | 1.82e-02 | 0.16 |  |
| Interaction effect of fit to wellbeing tasks | 1.82e-02 | 2.36e-02 | 0.44 |  |
| Interaction effect of sharing gratitude letters in intervention phase | -0.11 | 6.02e-02 | 8.11e-02 |  |
| Interaction effect of self-reported effort in intervention phase | 8.04e-03 | 2.45e-02 | 0.74 |  |
| Interaction effect of task effort in in intervention phase | 3.19e-02 | 2.84e-02 | 0.26 |  |
| *Follow-up phase intervention effects:* |  |  |  |  |
| Interaction effect of sex in follow-up phase | 0.19 | 6.75e-02 | 4.74e-03 |  |
| Interaction effect of year 1 SES in follow-up phase | 2.53e-02 | 3.04e-02 | 0.41 |  |
| Interaction effect of study wave in follow-up phase | -0.13 | 7.25e-02 | 7.99e-02 |  |
| Interaction effect of conscientiousness in follow-up phase | -2.63e-02 | 2.56e-02 | 0.30 |  |
| Interaction effect of motivation to becoming happier in follow-up phase | 9.88e-03 | 2.35e-02 | 0.67 |  |
| Interaction effect of sharing gratitude letters in follow-up phase | -5.23e-02 | 7.34e-02 | 0.48 |  |
| **Random Effects** | **SD** | | | |
| Level 1: |  | | | |
| Residual (e_i_) | 0.23 | | | |
| Level 2: |  | | | |
| Intercept | 0.22 | | | |
| Control phase | 0.24 | | | |
| Intervention phase | 0.16 | | | |
| Follow-up phase | 0.30 | | | |
| Level 3: |  | | | |
| (Intercept, U_0_) | 0.55 | | | |
| Control phase (U_1_) | 0.63 | | | |
| Intervention phase (U_2_) | 0.59 | | | |
| Follow-up phase (U_3_) | 0.65 | | | |
| AIC | 5158.04 | | | |
| BIC | 5562.40 | | | |
| logLik | -2510.02 | | | |

N= 648 twins in 358 families, 2592 observations

*Note*. This is a piecewise hierarchical linear mixed effects model for predicting changes in mental health and potential level 2 predictors of individual differences in response. Results show coefficients for all main and interaction effects. The 3 levels of the model incorporate repeated measures nested in twins nested in families.

There are fewer observations used in this model compared to the basic model. This is because multilevel modeling cannot account for missing values in predictor variables so the analysis has excluded cases that have missing values in any of the relevant predictors.

The basic model was rerun with the same participants as used in for the interaction model. Results from basic model with fewer observations were comparable with the original basic model.
