# Supplementary material for: Moderators of wellbeing interventions: Why do some people respond more positively than others?
Source: PLoS One. 2017 Nov 6;12(11):e0187601. doi: 10.1371/journal.pone.0187601 (PMC5673222; doi:10.1371/journal.pone.0187601)
Supplement: S12 Table — (DOCX) [file pone.0187601.s012.docx]

S12 Table. Fit statistics for mental health outcome models

| Model | df | AIC | BIC | logLik | Test | L.Ratio | p-value |
| --- | --- | --- | --- | --- | --- | --- | --- |
| 1. Intercept | 2 | 7164.61 | 7176.33 | -3580.31 |  | NA | NA |
| 1. Random Intercept | 3 | 5764.74 | 5782.32 | -2879.37 | 1 vs 2 | 1401.87 | 0 |
| 1. Random Intercept, 3 levels – repeated measures nested in twins nested in families | 4 | 5738.09 | 5761.54 | -2865.05 | 2 vs 3 | 28.65 | 8.69E-08 |
| 1. Random intercept and fixed slope predicted by time, 3 levels | 5 | 5710.82 | 5740.12 | -2850.41 | 3 vs 4 | 29.27 | 6.29E-08 |
| 1. Random intercept and 3 slopes (piecewise) predicted by 3 time phases, 3 levels | 7 | 5713.07 | 5754.09 | -2849.53 | 4 vs 5 | 1.76 | 0.42 |
| 1. Random intercept and 3 random slopes predicted by 3 time phases, 3 levels | 25 | 5693.50 | 5840.01 | -2821.75 | 5 vs 6 | 55.57 | 1.04E-05 |
| 1. Interaction model: individual slopes predicted by potential moderators, 3 levels | 69 | 5158.04 | 5562.40 | -2510.02 | 6 vs 7 | 623.46 | 0 |

*Note.* Table comparing the fit statistics of the fitted models for mental health as an outcome of intervention response. Models are built up from a simple intercept model to a full 3 level interaction model. Only cases which have complete data for all predictors used in the final interaction model are used in all models. Results show that each model is a significantly better fit for the data than the previous, other than model 5 compared to model 4. However, since we experimentally manipulated the study to have 3 separate phases, we decided to accept the piecewise model 5. Looking at the AIC values, subsequent models 6 and 7 are still better than model 4.
