# Supplementary material for: Moderators of wellbeing interventions: Why do some people respond more positively than others?
Source: PLoS One. 2017 Nov 6;12(11):e0187601. doi: 10.1371/journal.pone.0187601 (PMC5673222; doi:10.1371/journal.pone.0187601)
Supplement: S13 Table — (DOCX) [file pone.0187601.s013.docx]

S13 Table. Complete results for interaction model for wellbeing response, not including effort measures as predictors

| **Fixed parameter** | **Coefficient** | **SE** | ***p*-value** |  |
| --- | --- | --- | --- | --- |
| Intercept (β_0_) |  |  |  |  |
| γ_00_ | -4.53 | 0.20 | 1.14E-99 |  |
| Period 1, Control Phase (β_1_) |  |  |  |  |
| γ_10_ | 5.33E-02 | 0.11 | 0.63 |  |
| Period 2, Intervention Phase (β_2_) |  |  |  |  |
| γ_20_ | 0.13 | 0.13 | 0.31 |  |
| Period 3, Follow-up Phase (β_2_) |  |  |  |  |
| γ_30_ | 4.27E-02 | 3.76E-02 | 0.26 |  |
| Main effect of sex | -1.87E-02 | 4.92E-02 | 0.70 |  |
| Main effect of SES | -4.59E-02 | 2.39E-02 | 5.55E-02 |  |
| Main effect of study season | 4.05E-02 | 4.49E-02 | 0.37 |  |
| Main effect of extraversion | 9.49E-02 | 1.77E-02 | 1.45E-07 |  |
| Main effect of agreeableness | 6.89E-02 | 2.04E-02 | 8.03E-04 |  |
| Main effect of neuroticism | 0.21 | 1.76E-02 | 1.61E-28 |  |
| Main effect of sensation seeking | -7.67E-02 | 3.02E-02 | 1.16E-02 |  |
| Main effect of positive affect before intervention phase | 9.40E-02 | 7.43E-03 | 2.85E-30 |  |
| Main effect of gratitude before intervention phase | 0.27 | 2.77E-02 | 3.92E-20 |  |
| Main effect of prosociality before intervention phase | 6.34E-03 | 1.24E-02 | 0.61 |  |
| Main effect of hedonic adaptation to control tasks | -4.25E-02 | 1.56E-02 | 6.86E-03 |  |
| Main effect of hedonic adaption to wellbeing tasks | 3.13E-02 | 1.58E-02 | 4.85E-02 |  |
| Main effect of fit to wellbeing tasks | 6.81E-02 | 2.16E-02 | 1.73E-03 |  |
| *Control phase interaction effects:* |  |  |  |  |
| Interaction effect of sex in control phase | 8.60E-02 | 4.01E-02 | 3.21E-02 |  |
| Interaction effect of year 1 SES in control phase | -2.51E-02 | 1.84E-02 | 0.17 |  |
| Interaction effect of extraversion in control phase | 2.34E-02 | 1.44E-02 | 0.10 |  |
| Interaction effect of neuroticism in control phase | -1.14E-02 | 1.40E-02 | 0.42 |  |
| Interaction effect of sensation seeking in control phase | -3.30E-02 | 2.47E-02 | 0.18 |  |
| Interaction effect of hedonic adaptation to control tasks | -5.68E-03 | 1.22E-02 | 0.64 |  |
| *Intervention phase interaction effects:* |  |  |  |  |
| Interaction effect of sex in intervention phase | -4.90E-02 | 3.89E-02 | 0.21 |  |
| Interaction effect of study wave in intervention phase | 4.50E-02 | 3.82E-02 | 0.24 |  |
| Interaction effect of agreeableness in intervention phase | 1.54E-02 | 1.61E-02 | 0.34 |  |
| Interaction effect of positive affect before intervention phase | -9.82E-03 | 5.74E-03 | 8.74E-02 |  |
| Interaction effect of gratitude before intervention phase | -2.51E-02 | 2.16E-02 | 0.25 |  |
| Interaction effect of prosociality before intervention phase | 1.98E-02 | 9.83E-03 | 4.38E-02 |  |
| Interaction effect of hedonic adaptation to wellbeing tasks | -2.27E-02 | 1.19E-02 | 5.68E-02 |  |
| Interaction effect of fit to wellbeing tasks | 9.31E-03 | 1.71E-02 | 0.59 |  |
| *Follow-up phase interaction effects:* |  |  |  |  |
| Interaction effect of sex in follow-up phase | 0.11 | 3.96E-02 | 6.21E-03 |  |
| Interaction effect of year 1 SES in follow-up phase | 1.85E-02 | 1.74E-02 | 0.29 |  |
| Interaction effect of study season in follow-up phase | -8.70E-02 | 4.22E-02 | 3.92E-02 |  |
| **Random effects** | **SD** | | | |
| Level 1: |  | | | |
| Level 1 error | 0.11 | | | |
| Level 2: |  | | | |
| Intercept | 0.25 | | | |
| Control phase | 9.78e-02 | | | |
| Intervention phase | 8.03e-02 | | | |
| Follow-up phase | 0.12 | | | |
| Level 3: |  | | | |
| Intercept | 0.49 | | | |
| Control phase | 0.47 | | | |
| Intervention phase | 0.47 | | | |
| Follow-up phase | 0.47 | | | |
| AIC | 4042.36 | | | |
| BIC | 4371.50 | | | |
| logLike | -1966.18 | | | |

*N*= 737 twins in 391 families, 2935 observations

*Note*. This is a piecewise hierarchical linear mixed effects model for predicting changes in wellbeing and potential level 2 predictors of individual differences in response. The 3 levels of the model incorporate repeated measures nested in twins nested in families.

Looking at the relevant predictors of the interaction model (according to S5 Table), self-reported effort and task effort were the two predictors that produced the most missing values. We decided to run our interaction analysis excluding self-reported and task effort as relevant predictors. Results from this interaction model produced no Bonferroni significant moderators (the original interaction model produced self-reported effort during the control phase as a Bonferroni significant moderator)
