# Supplementary material for: Moderators of wellbeing interventions: Why do some people respond more positively than others?
Source: PLoS One. 2017 Nov 6;12(11):e0187601. doi: 10.1371/journal.pone.0187601 (PMC5673222; doi:10.1371/journal.pone.0187601)
Supplement: S14 Table — (DOCX) [file pone.0187601.s014.docx]

S14 Table. Complete results for interaction model for mental health response, not including effort measures as predictors

| **Fixed parameter** | **Coefficient** | **SE** | ***p*-value** |  |
| --- | --- | --- | --- | --- |
| Intercept (β_0_) |  |  |  |  |
| γ_00_ | -3.74 | 0.22 | 1.69E-61 |  |
| Period 1, Control Phase (β_1_) |  |  |  |  |
| γ_10_ | 0.45 | 0.17 | 7.03E-03 |  |
| Period 2, Intervention Phase (β_2_) |  |  |  |  |
| γ_20_ | 0.11 | 0.20 | 0.57 |  |
| Period 3, Follow-up Phase (β_2_) |  |  |  |  |
| γ_30_ | 0.14 | 0.16 | 0.39 |  |
| Main effect of sex | 7.75E-02 | 5.50E-02 | 0.16 |  |
| Main effect of SES | -2.17E-02 | 2.69E-02 | 0.42 |  |
| Main effect of study season | -3.76E-02 | 5.52E-02 | 0.50 |  |
| Main effect of extraversion | -2.59E-02 | 2.08E-02 | 0.21 |  |
| Main effect of agreeableness | -2.07E-02 | 2.35E-02 | 0.38 |  |
| Main effect of conscientiousness | 4.54E-02 | 1.86E-02 | 1.50E-02 |  |
| Main effect of neuroticism | 0.27 | 2.12E-02 | 3.08E-31 |  |
| Main effect of positive affect before control phase | 7.70E-02 | 1.14E-02 | 5.44E-11 |  |
| Main effect of positive affect before intervention phase | 5.47E-02 | 9.12E-03 | 5.39E-09 |  |
| Main effect of gratitude before intervention phase | 0.20 | 3.24E-02 | 1.58E-09 |  |
| Main effect of hedonic adaptation to control tasks | -3.05E-02 | 1.83E-02 | 0.10 |  |
| Main effect of hedonic adaption to wellbeing tasks | 2.06E-02 | 1.81E-02 | 0.25 |  |
| Main effect of fit to wellbeing tasks | -3.90E-02 | 2.43E-02 | 0.11 |  |
| Main effect of motivation to becoming happier | -6.52E-03 | 2.16E-02 | 0.76 |  |
| Main effect of sharing letter | -6.12E-02 | 5.03E-02 | 0.22 |  |
| *Control phase interaction effects:* |  |  |  |  |
| Interaction effect of sex in control phase | 9.31E-02 | 6.16E-02 | 0.13 |  |
| Interaction effect of year 1 SES in control phase | -6.26E-02 | 2.78E-02 | 2.43E-02 |  |
| Interaction effect of study wave in control phase | 7.96E-02 | 6.28E-02 | 0.20 |  |
| Interaction effect of extraversion in control phase | 4.71E-03 | 2.09E-02 | 0.82 |  |
| Interaction effect of neuroticism in control phase | -2.95E-02 | 2.32E-02 | 0.20 |  |
| Interaction effect of initial positive affect before control phase | -2.94E-02 | 1.06E-02 | 5.62e-03 |  |
| Interaction effect of hedonic adaptation to control tasks | -1.57E-02 | 1.76E-02 | 0.37 |  |
| *Intervention phase interaction effects:* |  |  |  |  |
| Interaction effect of sex in intervention phase | -0.12 | 5.71E-02 | 0.03 |  |
| Interaction effect of study wave in intervention phase | 8.91E-02 | 5.78E-02 | 0.12 |  |
| Interaction effect of agreeableness in intervention phase | 4.99E-02 | 2.19E-02 | 2.25E-02 |  |
| Interaction effect of conscientiousness in intervention phase | 1.93E-02 | 2.03E-02 | 0.34 |  |
| Interaction effect of neuroticism in intervention phase | -3.61E-02 | 1.89E-02 | 5.60E-02 |  |
| Interaction effect of initial positive affect before intervention phase | -1.19E-02 | 8.16E-03 | 0.14 |  |
| Interaction effect of initial gratitude before intervention phase | -1.44E-02 | 3.00E-02 | 0.63 |  |
| Interaction effect of hedonic adaptation to wellbeing tasks | -2.31E-02 | 1.62E-02 | 0.16 |  |
| Interaction effect of fit to wellbeing tasks | 2.19E-02 | 2.20E-02 | 0.32 |  |
| Interaction effect of sharing gratitude letters in intervention phase | -8.94E-02 | 5.59E-02 | 0.11 |  |
| *Follow-up phase interaction effects:* |  |  |  |  |
| Interaction effect of sex in follow-up phase | 0.16 | 6.42E-02 | 0.01 |  |
| Interaction effect of year 1 SES in follow-up phase | 2.27E-02 | 2.87E-02 | 0.43 |  |
| Interaction effect of study wave in follow-up phase | -9.32E-02 | 6.81E-02 | 0.17 |  |
| Interaction effect of conscientiousness in follow-up phase | -2.34E-02 | 2.43E-02 | 0.33 |  |
| Interaction effect of motivation to becoming happier in follow-up phase | 5.12E-03 | 2.21E-02 | 0.82 |  |
| Interaction effect of sharing gratitude letters in follow-up phase | -6.84E-02 | 6.88E-02 | 0.32 |  |
| **Random effects** | **SD** | | | |
| Level 1: |  | | | |
| Level 1 error | 0.26 | | | |
| Level 2: |  | | | |
| Intercept | 0.21 | | | |
| Control phase | 0.23 | | | |
| Intervention phase | 0.19 | | | |
| Follow-up phase | 0.31 | | | |
| Level 3: |  | | | |
| Intercept | 0.55 | | | |
| Control phase | 0.60 | | | |
| Intervention phase | 0.62 | | | |
| Follow-up phase | 0.26 | | | |
| AIC | 5745.55 | | | |
| BIC | 6121.73 | | | |
| logLike | -2809.78 | | | |

*N*= 724 twins in 385 families, 2896 observations

*Note*. This is a piecewise hierarchical linear mixed effects model for predicting changes in mental health and potential level 2 predictors of individual differences in response. The 3 levels of the model incorporate repeated measures nested in twins nested in families.

Looking at the relevant predictors of the interaction model (according to S10 Table), self-reported effort and task effort were the two predictors that produced the most missing values. We decided to run our interaction analysis excluding self-reported and task effort as relevant predictors. Results from this interaction model produced positive affect before the control phase as the predictor that approaches Bonferroni significance, which is comparable with the original interaction model.
