# Supplementary material for: Moderators of wellbeing interventions: Why do some people respond more positively than others?
Source: PLoS One. 2017 Nov 6;12(11):e0187601. doi: 10.1371/journal.pone.0187601 (PMC5673222; doi:10.1371/journal.pone.0187601)
Supplement: S1 Fig — (DOCX) [file pone.0187601.s015.docx]

S1 Fig. Power curve of interaction effect of agreeableness during the intervention phase for wellbeing outcome (with 95% confidence interval)


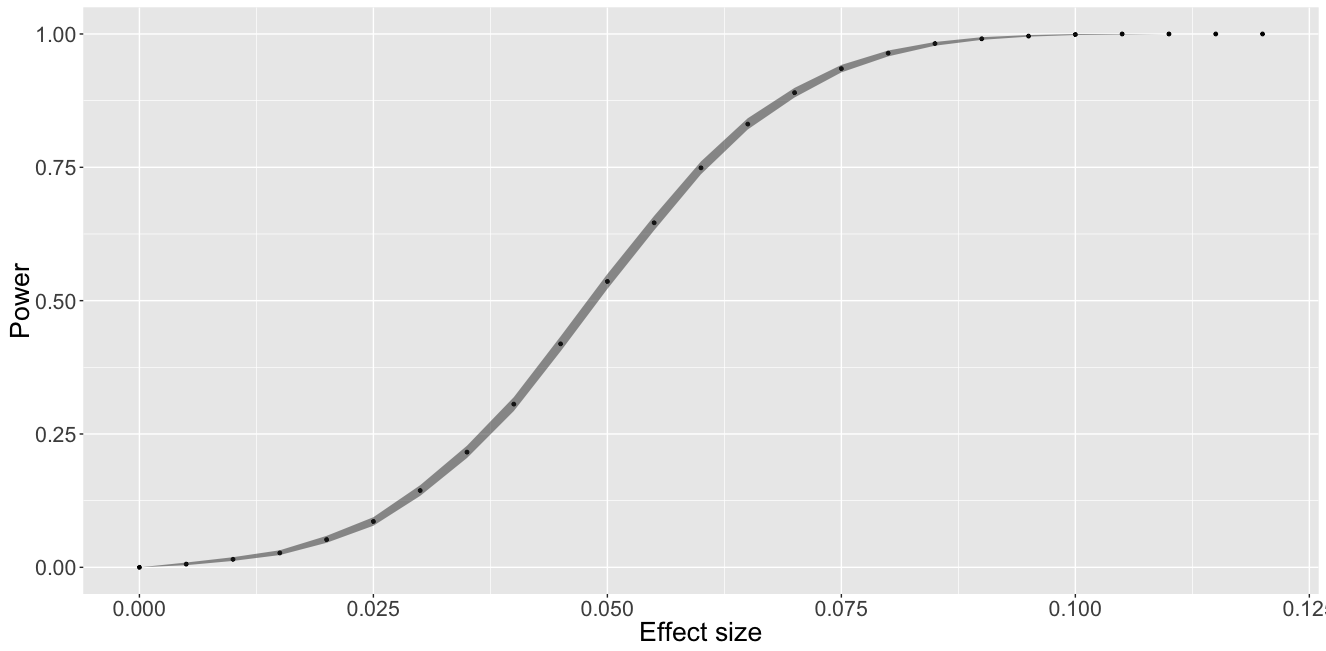


*Note*. Power curve showing power as a function of effect sizes for a constant sample of 360 families, each with 2 twins. These effect sizes are the interaction effects of personality trait agreeableness on wellbeing outcome during the intervention phase. The curve shows we have 80% power to detect an effect size of approximately 0.063.

Power analysis was conducted on MLPowSim using our defined random effect models. We used a Bonferroni corrected alpha of 0.0025. Number of simulations for each estimate = 10,000. We used agreeableness as it was the first in the alphabet of our list of moderators tested in our final interaction model.

Reference: Browne WJ, Lahi MG, Parker RM. A guide to sample size calculations for random effect models via simulation and the MLPowSim software package. Bristol, United Kingdom: University of Bristol. 2009.
